# Anti-N-Methyl-D-Aspartate Receptor Encephalitis Associated With Clear Cell Renal Carcinoma: A Case Report

**DOI:** 10.3389/fonc.2020.00350

**Published:** 2020-03-27

**Authors:** Jianhua Yang, Bin Li, Xiaoquan Li, Zhaohui Lai

**Affiliations:** Department of Neurology, The Affiliated Ganzhou Hospital of Nanchang University, Ganzhou, China

**Keywords:** anti-N-methyl-D-aspartate receptor encephalitis, autoimmune encephalitis, paraneoplastic syndrome, clear cell renal cancer, recurrence, seizure

## Abstract

**Background:** Anti-N-methyl-D-aspartate receptor (NMDAR) encephalitis is a cause of autoimmune encephalitis and is characterized by epileptic seizures, psychosis, and consciousness impairments. It mostly affects young adults with ovarian cancers. We herein reported a case of anti-NMDAR encephalitis associated with clear cell renal carcinoma.

**Case Presentation:** A 54-year-old male with headache for 1 week and mood and behavioral changes for 3 days was presented, but his clinical presentation and poor response to antiviral treatment did not support a diagnosis of viral encephalitis. Positive anti-NMDAR antibodies in serum and cerebrospinal fluid confirmed autoimmune encephalitis. A subsequent evaluation revealed a paraneoplastic etiology of a renal mass, and this was then resected and pathologically confirmed as clear cell renal carcinoma. The patient's symptoms showed improvement after resection of the mass. The patient relapsed 6 months after discharge, and the symptoms completely disappeared after treatment with corticosteroids and intravenous immunoglobulin.

**Conclusion:** Our findings suggested that NMDAR encephalitis might be associated with clear cell renal carcinoma. When patients present with unexplained seizures, neuropsychiatric disorder, or other brain symptoms, clinicians should be careful with paraneoplastic neurological disorders. Early diagnosis and treatment of primary tumors might show improvement.

## Introduction

Autoimmune encephalitis was first described more than 50 years ago (1), and a flood of novel clinical syndromes associated with neuronal autoantibodies has been recognized in the past 10 years. Anti-N-methyl-D-aspartate receptor (anti-NMDAR) encephalitis is mostly characterized by psychosis and memory impairment with abnormal movements in the early stage, and seizures and a depressed level of consciousness emerge as latter symptoms. Anti-NMDA receptor encephalitis is diagnosed by a blood or cerebrospinal fluid (CSF) test, imaging techniques, and electroencephalography (EEG). The identification of NMDAR antibodies in CSF or serum is currently the mainstay of diagnosis (2, 3). Magnetic resonance imaging (MRI) and CSF analysis might appear normal during the early stage of the disease. MRI, computed tomography (CT), and pelvic and transvaginal ultrasounds are necessary to confirm the underlying malignancies if NMDAR encephalitis is suspected (4). To date there are no estimates regarding the prevalence rates, but more than 500 cases have been reported, and anti-NMDA receptor encephalitis was found in the reproductive system of women with ovarian cancers in most of these cases (5). Cases have also been reported in older patients, predominantly men, but cancer is less frequent in this age range. Tumors other than teratoma are rare. We report here a case of anti-NMDAR encephalitis associated with clear cell renal cancer.

The removal of underlying tumors and subsequent immunotherapy with corticosteroids, intravenous immunoglobulins (IVIg), or plasma exchange were considered as first-line treatment, and rituximab or cyclophosphamide was used as second-line treatment (4). About 75% of patients with NMDAR antibodies benefited from tumor removal and immunotherapy, which thus resulted in full recovery or having a mild sequelae, while other patients were either severely disabled or died (2).

## Case Presentation

A 54-year-old male farmer belonging to Han nationality, with unremarkable medical history, was admitted to the Affiliated Ganzhou Hospital of Nanchang University, Department of Neurology, on April 9, 2017 for evaluation of headache for 1 week and mood and behavioral changes for 3 days. A neurological examination revealed mental disorder including dysphoria, excitement, gibberish, and nuchal rigidity. EEG and cerebral MRI revealed normal results. Lumbar puncture was then performed. A CSF analysis demonstrated leukocytic pleocytosis of 80,000 cells/ml, glucose levels of 4.76 mmol/L (normal range, 2.8–4.5 mmol/L), and protein levels of 16.4 mg/dl (normal range, 8–43 mg/dl). Antiviral treatment with acyclovir (0.5 g per 8 h, intravenously, for 3 days) was initiated for viral encephalitis, but the symptoms showed no improvement. He became agitated, confused, and disoriented 3 days after admission. Mildazolam (0.8 mg/h, intravenously) was used as a sedative treatment. On April 12, 2017, another lumbar puncture was performed, which showed positive anti-NMDAR antibodies in the serum as well as in CSF (antibody titer of 1:32) and negative anti-AMPA1, AMPA2, LGI1, Caspr2, and GABA-B antibodies (the antibody testing was performed by Kindstar Global Company). These diagnostic tests confirmed anti-NMDAR encephalitis. High doses of corticosteroids (methylprednisolone, 1 g/day for 3 days) and intravenous immunoglobulin (0.4 g/kg body weight) were administered.

**Figure 1:**
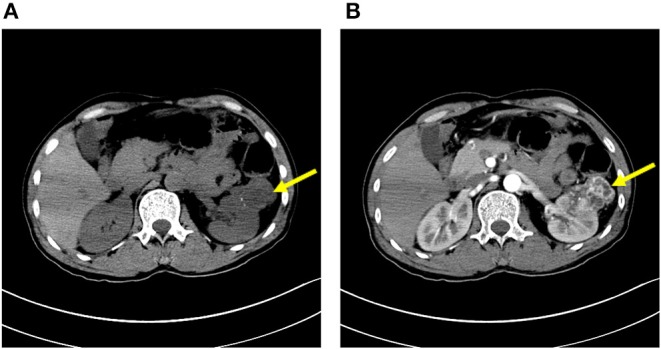
Plain CT **(A)** and contrast-enhanced scan **(B)** of the kidney. The arrow shows a mass of 5.5 × 5.0 × 4.0 cm on the left side.

**Figure 2:**
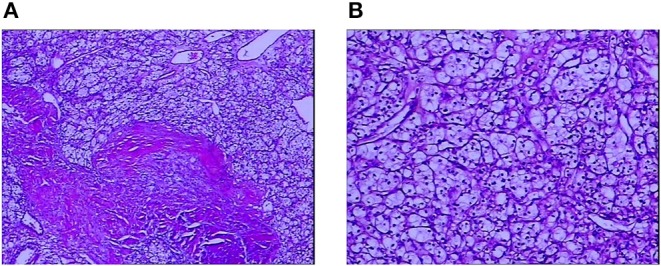
A pathological study of the mass on the left kidney confirmed it as renal cell carcinoma, clear cell type. **(A)** Low magnification. **(B)** High magnification. The cells have a clear cytoplasm, surrounded by a distinct cell membrane, and contain round and uniform nuclei.

As autoimmune encephalitis is cancer-related, CT scans of the chest, the abdomen, and the pelvis were performed on April 19, 2017, which revealed an exophytic mass on the left kidney and enhanced abdomen CT scans confirmed it as clear cell renal carcinoma (Figure 1). Laparoscopic partial nephrectomy was then performed with tracheal intubation and mechanical ventilation. The patient then had pneumonia and pleural effusion as surgical complications, so he was transferred to the intensive care unit. The pathology report showed renal cell carcinoma, clear cell type, and Fuhrman grade of 2, 5.5 × 5.0 × 4.0 cm (Figure 2). After resection of the mass, the sedative drugs and intubation were discontinued. After corticosteroid (1 g per day, intravenously, for 5 days, 500 mg per day for 3 days, 250 mg per day for 3 days) and cyclophosphamide (0.4 g, intravenously, once a week for a month) treatment, his consciousness was significantly improved, and neurophysiologic examination, EEG, and cerebral MRI were completely unremarkable; the patient was discharged after 1 month of hospitalization. He was hospitalized again due to mood and behavioral changes on October 15, 2017. Lumbar puncture was performed, which showed that the titer of the anti-NMDAR antibody was 1:32 in CSF. MRI and routine CSF analysis appeared normal. Melthyprednisolone pulse therapy (1 g/day for 3 days) and intravenous immunoglobulin (0.4 g/kg body weight/day for 3 days) were then given. The symptoms of mood and behavioral changes then disappeared. Oral administration of prednisone (20 mg) was suggested after discharge. The patient was persuaded to take the medicine every day. The titer of the anti-NMDAR antibody was decreased to 1:1 in CSF on June 26, 2018 and medication was discontinued. The patient remained free from recurrence for 11 months without receiving any medication. The patient was also advised to regularly observe follow-up visits to his doctor. His last visit was on December 12, 2018, and he had normal mental status.

## Discussion

We reported a case of anti-NMDAR encephalitis associated with clear cell renal carcinoma. The case reported herein had a classical clinical pattern of anti-NMDAR encephalitis characterized by initial psychiatric symptoms followed by loss of consciousness within a few weeks. The brain MRI and EEG appeared normal, while the anti-NMDAR antibodies were detected in serum as well as in CSF. However, this case showed several unusual features. Firstly, a 54-year-old male was affected with encephalitis. Generally, NMDAR encephalitis occurs in young adults and children with or without teratomas (2, 6). Secondly, the associated tumor was clear cell renal cancer. Finally, the patient was cured after undergoing immunotherapy and tumor resection. To our knowledge, this is the first report with regard to the detection of NMDAR antibodies in clear cell cancer.

Renal cell carcinoma is not usually associated with paraneoplastic neurological syndromes (PNS), and its association with autoimmune encephalitis is rare (7). Few cases with limbic encephalitis in association with renal cell carcinoma have been reported (5), but no autoimmune antibodies in serum or CSF were detected in these cases, so this is the first case of NMDAR encephalitis associated with renal cell carcinoma, which was cured with nephron-sparing surgery.

Anti-NMDAR encephalitis is an immune-mediated disorder that is associated with IgG antibodies against the GluN1 subunit of NMDAR (2). It was first described by Dalmau in 2007 and shown to have an association with ovarian teratoma in a young woman (8). The understanding of this disorder has greatly increased and more cases have been reported since its initial description in 2007; however, clinicians in this field are unaware of the disease features. It is vital for psychiatrists to be aware of this condition in a wide age spectrum and to contact neurology colleagues more promptly, thus facilitating early screening and diagnosis.

The pathological relationship between renal carcinoma and production of pathogenic autoantibodies (NMDA-R antibodies) remains unclear. The anti-neuronal antibodies associated with PNS are divided into two broad categories: antibodies that target intracellular neuronal antigens that are expressed by the cancer and the other antibodies that target proteins or receptors residing on neuronal cell surface or in the synapse. The NMDA-R antibody belongs to the latter category, and it is the antibody that mediates neuronal dysfunction by direct interaction with target antigens in the central nervous system (9). Thus, the patients often fully recovered or have shown significant improvement with immunotherapy or tumor resection if present. This presents the opportunity for early diagnosis and early treatment and it is clear that early identification and treatment might have serious prognostic implications. Delayed treatment with immunosuppressive therapy probably results in worse outcomes.

Ovarian teratoma is most commonly reported in young females. Besides tumors of the reproductive system, NMDAR encephalitis also showed an association with hepatic carcinoma, small cell lung carcinoma, thymic carcinoma, pancreatic cancer, breast cancer, Hodgkin's lymphoma, etc. (2, 4, 10, 11). According to the previous literature, anti-NMDAR encephalitis occurs in association with any neuroendocrine carcinoma. Hepatic and renal carcinomas are solid cancer types. Compared with hepatic carcinoma (11), both patients presented with psychiatric manifestations, but no epilepsy and motor manifestations occurred, which improved with early immunotherapy. This might be due to the fact that the patients were either diagnosed early with access to early treatment or had expressed a milder form of disorders. In order to clarify the relationship between tumor antigen exposure and anti-NMDAR encephalitis, it should bring benefit to conduct experiments that would determine which subunit of the NMDAR, N1 or N2b, is expressed in tumor tissues. However, we have not tested the NMDAR subunit in this patient yet, which is a limitation of this case.

PNS is an important tumor biomarker of renal cell carcinoma. The recognition and the early management of PNS can improve the prognosis of patients, although its underlying pathomechanisms are not fully understood. Physicians should assist in improving the understanding of this disease.

This case adds to the current literature of yet another tumor association (renal neuroendocrine carcinoma) of anti-NMDAR encephalitis in an elderly person. This case suggests that when patients present with unexplained seizures, neuropsychiatric disorder, or other brain symptoms, it is crucial for clinicians to be aware of PNS, so it is necessary to evaluate the presence of NMDAR antibodies and perform CT scans of the chest, the abdomen, and the pelvis when autoimmune encephalitis is suspected.

## Data Availability Statement

The datasets used/analyzed during the current study are available from the corresponding author on reasonable request.

## Ethics Statement

The studies involving human participants were reviewed and approved by Affiliated Ganzhou Hospital of Nanchang University. The patients/participants provided their written informed consent to participate in this study and to the publication of this case report.

## Author Contributions

JY and BL carried out the studies, participated in collecting data, and drafted the manuscript. XL participated in its design. ZL participated in the acquisition and analysis or interpretation of data. All authors read and approved the final manuscript.

### Conflict of Interest

The authors declare that the research was conducted in the absence of any commercial or financial relationships that could be construed as a potential conflict of interest.
